# Micro-droplet-based calibration for quantitative elemental bioimaging by LA-ICPMS

**DOI:** 10.1007/s00216-021-03357-w

**Published:** 2021-05-05

**Authors:** Andreas Schweikert, Sarah Theiner, Debora Wernitznig, Anna Schoeberl, Martin Schaier, Sophie Neumayer, Bernhard K. Keppler, Gunda Koellensperger

**Affiliations:** 1 Institute of Analytical Chemistry, Faculty of Chemistry, University of Vienna, Waehringer Strasse 38, 1090 Vienna, Austria; 2 Institute of Inorganic Chemistry, Faculty of Chemistry, University of Vienna, Waehringer Strasse 42, 1090 Vienna, Austria

**Keywords:** Laser ablation, Mass spectrometry, ICP-MS, Quantification strategy, Bioimaging

## Abstract

In this work, a novel standardization strategy for quantitative elemental bioimaging is evaluated. More specifically, multi-element quantification by laser ablation-inductively coupled plasma-time-of-flight mass spectrometry (LA-ICP-TOFMS) is performed by multi-point calibration using gelatin-based micro-droplet standards and validated using in-house produced reference materials. Fully automated deposition of micro-droplets by micro-spotting ensured precise standard volumes of 400 ± 5 pL resulting in droplet sizes of around 200 μm in diameter. The small dimensions of the micro-droplet standards and the use of a low-dispersion laser ablation setup reduced the analysis time required for calibration by LA-ICPMS significantly. Therefore, as a key advance, high-throughput analysis (pixel acquisition rates of more than 200 Hz) enabled to establish imaging measurement sequences with quality control- and standardization samples comparable to solution-based quantification exercises by ICP-MS. Analytical figures of merit such as limit of detection, precision, and accuracy of the calibration approach were assessed for platinum and for elements with biological key functions from the lower mass range (phosphorus, copper, and zinc). As a proof-of-concept application, the tool-set was employed to investigate the accumulation of metal-based anticancer drugs in multicellular tumor spheroid models at clinically relevant concentrations.

**Supplementary Information:**

The online version contains supplementary material available at 10.1007/s00216-021-03357-w.

**Figure:**
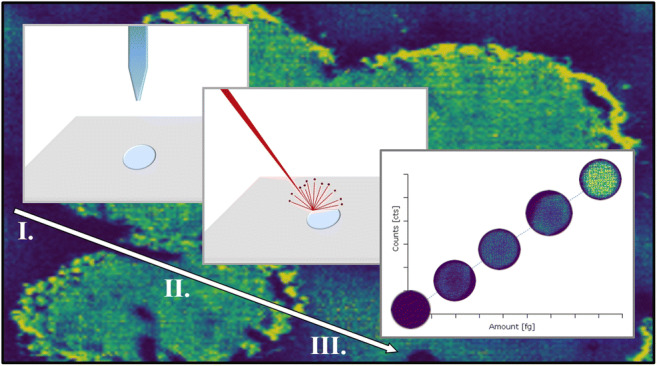
Graphical abstract

## Introduction

Laser ablation-inductively coupled plasma mass spectrometry (LA-ICPMS) has become an established technique for bioimaging applications as it is characterized by multi-element analysis capabilities, limits of detection at the sub-μg g^−1^ level, and a high spatial resolution down to the low micrometer level. Not only the spatial distribution of metals in biological systems is of importance, also their concentration levels play an important role in biological responses. However, the lack of suitable and certified reference materials for bioimaging applications by LA-ICPMS impedes proper validation. Therefore, major emphasis has been put on the development of matrix-matched calibration standards. Depending on the application, tissue-type sections have been prepared as calibration standards using homogenates of e.g. liver/brain tissue/chicken breast/whole blood [1–4] that are spiked with varying concentrations of liquid standards. As a drawback, these procedures are laborious and involve the handling of biological material. In addition, this calibration method does not provide perfect matrix-matching as a result of the difference in texture of sample and standard and the possible influence that this may have on the laser-sample coupling. Therefore, quantification concepts have been proposed aiming at general suitability for bioimaging by LA-ICPMS. In this context, polymer-based calibration standards [5] and ink-printed standards using commercially available printers [6, 7] have been developed. Parallel solution-based ICP-MS analysis served for validation of the novel standardization strategies [8]. The potential of isotope dilution strategies was evaluated for LA-ICPMS analysis, which also enabled to determine measurement uncertainties [9, 10].

Alternatively, calibration based on universal biological matrix mimics was proposed resorting to gelatin as an external calibrant, resembling the composition of biological materials. Its properties partially resemble properties of the fibrous protein collagen and its protein content matches the protein-rich cellular material. It was shown that particle transport properties are similar for gelatin and for biological material [11]. Moreover, gelatin properties can be easily fine-tuned, no handling of biological material is required, and the concentration range and number of analytes can be easily adjusted. Gelatin sections [12, 13] as well as gelatin droplets [8, 14, 15] have been used for the preparation of calibration standards for LA-ICPMS analysis. In one study, a micro-array of gelatin standards was developed and the accumulation of Cu in a model algae organism was used as a case study. The quantitative cellular distribution of Cu was cross-validated by synchrotron radiation X-ray fluorescence [16].

In droplet-based approaches, elemental distributions can be heterogeneous due to the coffee stain effect and/or the Marangoni effect during the drying process [14]. It was shown that these effects can be circumvented by optimization of the drying/setting conditions of the gelatin droplets and that highly homogeneous multi-element calibration standards based on gelatin can be prepared [14]. Another possibility to overcome the problem of elemental inhomogeneity is the ablation of the entire droplet [17, 18]. One study evaluated both approaches by comparing quantitative ablation of dried gelatin droplets and spot ablation of homogeneous gelatin gels in combination with the determination of the ablated volume by atomic force microscopy [19]. Using both approaches, quantification of membranous receptors was performed in breast cancer cell lines by high-resolution LA-ICPMS using lanthanide-labeled tracers [19]. Quantitative ablation of entire droplets can be time-consuming due to the dimensions of dried droplets, especially when deposition of the droplets is performed manually. Using micropipettes, volumes of around 1–300 μL [17, 19] and droplet areas of up to 4.5–6 mm^2^ [18] have been reported for gelatin droplets. The droplet size can be reduced by drying the droplets on a hydrophobic (polymeric) surface or the analysis time can be reduced by sampling a representative part (cross-section) of the droplet. One method to decrease the droplet size and automate droplet deposition made use of a MicroFab Jetlab micro-dispensing unit to create a rectangular grid of gold standard solutions [20]. This approach was applied to a peptide-Au cluster in human erythroleukemia cells [20] and to single cells to quantify the number of uptaken Au nanoparticles (NPs) [21]. In another study, a modified commercial ink cartridge and dosing device was used to generate pL-droplet residues for calibration via standard addition. The developed approach allowed the quantitative elemental characterization of thin-layered materials by LA-ICPMS [22]. Alternatively, micro-array spotters have been used to produce calibration standards in an automated and reproducible way. ICP-MS standard solutions were printed on nitrocellulose membranes [23] and on target glass slides [24] with volumes in the picoliter range [23, 25]. In this regard, single-cell analysis by LA-ICPMS was performed to quantify an Ir-DNA intercalator and an mDOTA-Ho dye in adherent 3T3 fibroblast cells [23]. Calibration standards using Ag NP suspensions were prepared in a similar manner to investigate the quantitative distribution of Ag NPs in a multicellular tumor spheroid model [25].

This work describes a calibration approach based on gelatin micro-droplet standards generated by a micro-droplet spotter for multi-element quantification in bioimaging applications by LA-ICPMS. The laser ablation setup is based on the prototype COBALT low-dispersion ablation cell [26], which enables pixel-resolved imaging at repetition rates of > 200 Hz for biological samples [27]. For multi-element analysis, an ICP-TOFMS instrument is used, which provides analyte detection between *m*/*z* = 14–256. Therefore, the quantitative analysis of elements with biological key functions (e.g., P, Fe, Cu, and Zn) is addressed together with the quantification of non-endogenous elements from the higher mass range. As proof of principle, the micro-droplet quantification concept was applied to sections of multicellular tumor spheroids, which were treated with platinum-based chemotherapeutics at clinically relevant concentrations.

## Experimental

### Chemicals and reagents

Ultrapure water (18.2 MΩ cm, ELGA Water Purification System, Purelab Ultra MK 2, UK or 18.2 MΩ cm, Milli-Q Advantage, Darmstadt, Germany) and nitric acid (>69%, Rotipuran Supra, Carl Roth, Karlsruhe, Germany) were used for all dilutions for ICP-MS measurements. For digestion, H_2_O_2_ (30%, Suprapur, Merck, Darmstadt, Germany) was added. A multi-element stock solution and single-element standard solutions were purchased from LabKings (Hilversum, The Netherlands). Gelatin (from cold water fish skin) was obtained from Sigma-Aldrich (Vienna, Austria). Pooled human plasma was purchased from Dunn Labortechnik (Asbach, Germany). The serum reference material Seronorm (Seronorm Trace Elements Serum L-1, Norway) was reconstituted according to the manufacturer’s protocol. TM-28.4 Lake Ontario water certified reference material (Environment and Climate Change, Burlington, Canada) was used. Sample preparation and measurements were carried out in clean room classes 100.000 and 10.000, respectively. All cell culture media and reagents were purchased from Sigma-Aldrich (Vienna, Austria) and all plastic dishes, plates, and flasks from StarLab (Hamburg, Germany) unless stated otherwise. Cisplatin and oxaliplatin were synthesized at the Institute of Inorganic Chemistry, University of Vienna, according to literature procedures [28, 29].

### Preparation of calibration standards for LA-ICP-TOFMS analysis

A CellenONE X1 micro-spotter and cell arrayer (Cellenion, Lyon, France) was used to produce arrays of gelatin micro-droplets, containing multi-element standard solutions onto glass slides. The instrument is a contactless spotter generating droplets by use of a piezo crystal to induce shockwaves in the sample containing capillary and thereby producing droplets of 300 to 500 pL in volume (depending on the specific capillary used). The size of the droplets produced by the instrument is evaluated before and after each spot run visually by the software of the instrument. Droplets are spotted in an equidistance. The holder for the sample source and targets is cooled to 16 °C, and the humidity can be raised to reach the thaw point to prevent evaporation of the sample. Liquid multi-element standard solutions were prepared gravimetrically from commercial standard stock solutions in 1% (v/v) HNO_3_. Fish gelatin stock solution (10%, w/w) was added to reach a final concentration of 1% (w/w) gelatin. The resulting solutions were transferred into wells of a 384-well plate, which serves as the sample source for the micro-spotter. The system liquid of the micro-spotter was replaced daily by freshly filtered and degassed ultrapure water. The rinse solution was replaced daily by ultrapure water. The gelatin standards were spotted onto glass slides using the micro-array spotter with a droplet size of 400 ± 5 pL. Due to the very small volume of the droplets, they fully dry within seconds. The slides were stored at room temperature until LA-ICP-TOFMS analysis. The long-term stability of the micro-droplet gelatin standards was evaluated over a period of several months.

### Multi-element analysis of gelatin standards by ICP-MS

The concentrations of the investigated elements in the gelatin standards were confirmed using solution-based ICP-MS analysis. Aliquots of gelatin standards (30 μL) were weighed in PFA tubes, and 2 mL of 20% (v/v) HNO_3_ and 100 μL of 30% (v/v) H_2_O_2_ were added. The resulting solutions were put on a hot plate and a temperature program was run, heating the solutions for 6 h with a maximum temperature of 200 °C. After the liquids have cooled down, they were transferred to 15-mL tubes and filled up to 10 mL with ultrapure water. An Agilent 7800 quadrupole-based ICP-MS instrument (Agilent Technologies, Tokyo, Japan) was used for multi-element analysis of the gelatin standards. The ICP-MS instrument was equipped with an Agilent SPS 4 autosampler (Agilent Technologies, Tokyo, Japan), a MicroMist nebulizer at a sample uptake rate of approx. 200 μL min^−1^, and standard nickel cones. The instrument was tuned on a daily basis to achieve maximum sensitivity, low oxide formation (^140^Ce^16^O^+^/^140^Ce^+^ < 1.5%), and a low doubly charged ratio (^140^Ce^2+^/^140^Ce^+^ < 2%). Elements were monitored in standard mode and in helium gas mode with a gas flow rate of 3 mL min^−1^. The following nuclides with a respective integration time of 0.1 s were monitored in standard mode: ^31^P^+^, ^47^Ti^+^, ^55^Mn^+^, ^59^Co^+^, ^63^Cu^+^, ^75^As^+^, ^78^Se^+^, ^95^Mo^+^, ^121^Sb^+^, ^137^Ba^+^, and ^195^Pt^+^. The following nuclides with a respective integration time of 0.1 s were monitored in helium gas mode: ^24^Mg^+^, ^51^V^+^, ^52^Cr^+^, ^56^Fe^+^, ^60^Ni^+^, ^66^Zn^+^, ^111^Cd^+^, ^205^Tl^+^, and ^208^Pb^+^. Indium and rhenium were used as internal standards. The TM-28.4 Lake Ontario water certified reference material (Environment and Climate Change, Burlington, Canada) was used to verify the accuracy of the measurement. The Agilent MassHunter software package (Workstation Software, version C.01.04, 2018) was used for data evaluation. The instrumental parameters of the ICP-MS measurements are summarized in Table S1 in Supplementary Information (ESM).

### Multi-element analysis of spiked human plasma samples by FI-ICP-MS/MS

Human plasma samples were spiked with varying concentrations of P, Fe, Cu, Zn, and Pt elemental standard solutions. Due to the acidity of the standard solutions, the concentrations were kept in a range where no protein precipitation of the plasma was observed. The spiked plasma samples and Seronorm serum reference material were spotted onto glass slides using the micro-array spotter similarly to the gelatin standard droplets with a droplet volume of 400 ± 5 pL and stored at room temperature until LA-ICP-TOFMS analysis. An Agilent 8800 quadrupole-based ICP-MS/MS instrument (Agilent Technologies, Tokyo, Japan) was used for flow injection (FI) analysis of the spiked human plasma samples. The sample introduction system consisted of a MicroMist nebulizer (200 μL min^−1^ nominal uptake, AHF Analysentechnik AG, Tuebingen, Germany) and a quartz double-pass spray chamber (Agilent Technologies, Waldbronn, Germany). The instrument was tuned on a daily basis to achieve maximum sensitivity, low oxide formation (^140^Ce^16^O^+^/^140^Ce^+^ < 1.5%), and a low doubly charged ratio (^140^Ce^2+^/^140^Ce^+^ < 2%). The following isotopes with a respective integration time of 0.1 s were monitored in standard mode: ^63^Cu^+^, ^64^Zn^+^, ^65^Cu^+^, ^66^Zn^+^, and ^195^Pt^+^. The following isotopes with a respective integration time of 0.1 s were monitored in oxygen gas mode (with a gas flow rate of 0.30 mL min^−1^) and mass selection steps on both quadrupoles: ^31^P^+^ → ^31^P^16^O^+^ and ^56^Fe^+^ → ^56^Fe^16^O^+^. The accuracy of the FI-ICP-MS/MS measurement was determined using Seronorm serum reference material (Seronorm Trace Elements Serum L-1, Norway). A bio-inert Agilent 1260 HPLC system (Agilent Technologies, Waldbronn, Germany) was used for flow injection measurements. The HPLC was directly connected to the nebulizer of the ICP-MS/MS system by a PEEK capillary tubing. The following chromatographic conditions were used: injection volume: 5 μL; flow rate: 0.30 mL min^−1^; isocratic elution; CH_3_COONH_4_ (50 mM, pH = 6.8) was employed as a mobile phase. The autosampler of the HPLC system was cooled to 4 °C. The data were recorded and evaluated with the Agilent MassHunter Chromatography software package (MassHunter 4.1, version C.01.01, 2014). Instrumental parameters for ICP-MS/MS measurements are summarized in ESM Table S1.

### Tumor spheroids

The human colon carcinoma cell line HCT-116 was obtained from ATCC (VA, USA) and was maintained in McCoy’s 5a supplemented with 10% fetal calf serum (FCS) and l-glutamine. All cells were cultured as adherent monolayers in 75-cm^2^ flasks and kept in a humidified incubator at 37 °C with 5% CO_2_. For spheroid generation, HCT-116 cells were harvested from culture flasks by trypsinization, re-suspended in supplemented medium, and seeded in ultra-low attachment round-bottom 96-well plates (Nunclon Sphera™, Thermo Fisher Scientific) at a density of 10.000 viable cells per well. Plates were incubated at 37 °C with 5% CO_2_ for 96 h to allow spheroid formation and then used for the experiments. Both compounds were dissolved in McCoy’s 5a and stock solutions were diluted stepwise to obtain the final concentrations. In total, 100 μL of the dilution was added to each well, and treated plates were incubated for 12 and 24 h at 37 °C with 5% CO_2_. Spheroids were collected, pooled, and embedded in TissueTek (Sakura). Samples were frozen at −80 °C until further processing. Samples were cut into sections of 5-μm thickness using a cryostat (Leica) and placed onto Superfrost slides (Thermo Scientific) for LA-ICP-TOFMS analysis. The sections were dried at room temperature before measurement.

### LA-ICP-TOFMS analysis

An Analyte Excite Excimer 193-nm laser ablation system (Teledyne Photon Machines, Bozeman, MT, USA) was coupled to an *icp*TOF 2R (TOFWERK AG, Thun, Switzerland) TOF-based ICP-MS instrument. The laser ablation system was equipped with a prototype tube-type COBALT ablation cell [26] and the aerosol rapid introduction system (ARIS). Through the low-dispersion mixing bulb of the ARIS, an Ar make-up gas flow (~0.90 L min^−1^) was introduced into the optimized He carrier gas flow (0.50 L min^−1^) before entering the plasma. The LA and ICP-TOFMS settings were optimized on a daily basis while ablating NIST SRM612 glass certified reference material (National Institute of Standards and Technology, Gaithersburg, MD, USA). Optimization was based on high intensities for ^24^Mg^+^, ^59^Co^+^, ^115^In^+^, and ^238^U^+^; low oxide formation based on the ^238^U^16^O^+^/^238^U^+^ ratio (<2%); and low elemental fractionation based on the ^238^U^+^/^232^Th^+^ ratio (~1). Laser ablation sampling was performed in fixed dosage mode 2, at a repetition rate of 200 Hz and using a 5 μm × 5 μm square spot. The line scans overlapped one another by 2.5 μm and the used laser ablation parameters resulted in a pixel size of 2.5 μm × 2.5 μm. Selective ablation of the gelatin micro-droplets, plasma droplets, and tumor spheroids was achieved by selecting an energy density below the ablation threshold of glass and above the ablation threshold of gelatin and the samples. Gelatin micro-droplets and biological samples were removed quantitatively using a fluence of 0.60–0.90 J cm^−2^ [30].

The *icp*TOF 2R ICP-TOFMS instrument has a specified mass resolution (*R* = *m/*Δ*m*) of 6000 (full width half-maximum definition). The standard operation mode was used, which balances mass resolving power, sensitivity, and ion transmission across the entire measured mass range and which allows the analysis of ions from *m*/*z* = 14–256. The integration and read-out rate match the LA repetition rate. The instrument was equipped with a torch injector of 2.5-mm inner diameter and nickel sample and skimmer cones with a skimmer cone insert of 2.8 mm in diameter. A radio frequency power of 1440 W, an auxiliary Ar gas flow rate of ~0.80 L min^−1^, and a plasma Ar gas flow rate of 14 L min^−1^ were used. In case the CCT mode was used, the collision/reaction cell was pressurized with a mixture of H_2_/He gas with an optimized flow rate of 2.0 mL min^−1^. The following CCT parameters were used: CCT focus lens: −1 V, CCT entry lens: −75 V, CCT mass: 230 V, CCT bias: 3 V, CCT exit lens: −160 V [31]. Instrumental parameters for LA-ICP-TOFMS measurements are summarized in ESM Table S1.

### Data acquisition and processing of LA-ICP-TOFMS data

Data was recorded using TofPilot 1.3.4.0 (TOFWERK AG, Thun, Switzerland). The LA-ICP-TOFMS data were saved in the open-source hierarchical data format (HDF5, www.hdfgroup.org). Post-acquisition data processing was performed with Tofware v3.2.0, which is a TOFWERK data analysis package and used as an add-on on IgorPro (Wavemetric Inc., OR, USA). The data processing comprised the following steps: (1) drift correction of the mass peak position in the spectra over time via time-dependent mass calibration, (2) determining the peak shape, and (3) fitting and subtracting the mass spectral baseline. The data was further processed with HDIP version 1.3.1.1038 (Teledyne Photon Machines, Bozeman, MT, USA). An integrated script was used to automatically process the files generated by Tofware and to generate 2D elemental distribution maps. For calibration, signal responses for each mass channel monitored during ablation of a single spiked droplet were integrated using HDIP. The sum of the elemental signal intensities and the absolute masses of the respective element within the droplet were used to set up calibration curves. In order to obtain elemental concentrations for each pixel of the tumor spheroid samples, the total ablated tissue mass per pixel (*m*_*p*_) can be calculated based on the tissue thickness prior to drying (*δ*_*t*_), the ablated area (*a*^2^) per pixel, and the tissue density (*ρ*_*t*_). Equations for all calculations are summarized in the ESM (Equations S1–S8).

## Results and discussion

### Multi-element calibration using gelatin micro-droplets and LA-ICP-TOFMS analysis

A calibration approach based on a micro-array spotter was developed and optimized to generate multi-element standards for bioimaging applications by LA-ICP-(TOF)MS. The use of a non-contact micro-droplet dispenser enabled the spotting of low volumes of 400 ± 5 pL. This resulted in droplet sizes of around 150–200 μm in diameter with known volumes for every droplet standard concentration. Gelatin from cold water fish skin was selected as a matrix because it does not solidify at ambient temperature, thereby avoiding blockage of the capillary. The used micro-droplet dispenser allowed spotting of droplets on up to four glass slides in a single run. Spotting four glass slides with 10 different standards and a spot count of about 1500 per slide resulted in an operation time of around 1 h. The small volume of the droplets allowed them to dry within a few seconds and the calibration standards were stable at room temperature.

Droplets can show heterogeneous elemental distributions due to the coffee stain effect and/or the Marangoni effect [14]. Therefore, the ablation of the entire droplet standards was performed using the same parameters as for the samples. Elemental distribution maps of selected elements in micro-droplet gelatin standards are shown in ESM Fig. S2. The reduced dimensions of the droplets (compared to manual pipetting) in combination with the pixel acquisition rates provided by the used low-dispersion LA setup resulted in an analysis time of about 5 min for one entire gelatin micro-droplet standard. Therefore, imaging measurement sequences including standardization and quality control samples can be established for LA-ICP-MS analysis which are comparable to multi-element quantification by solution-based ICP-MS.

**Fig. 1:**
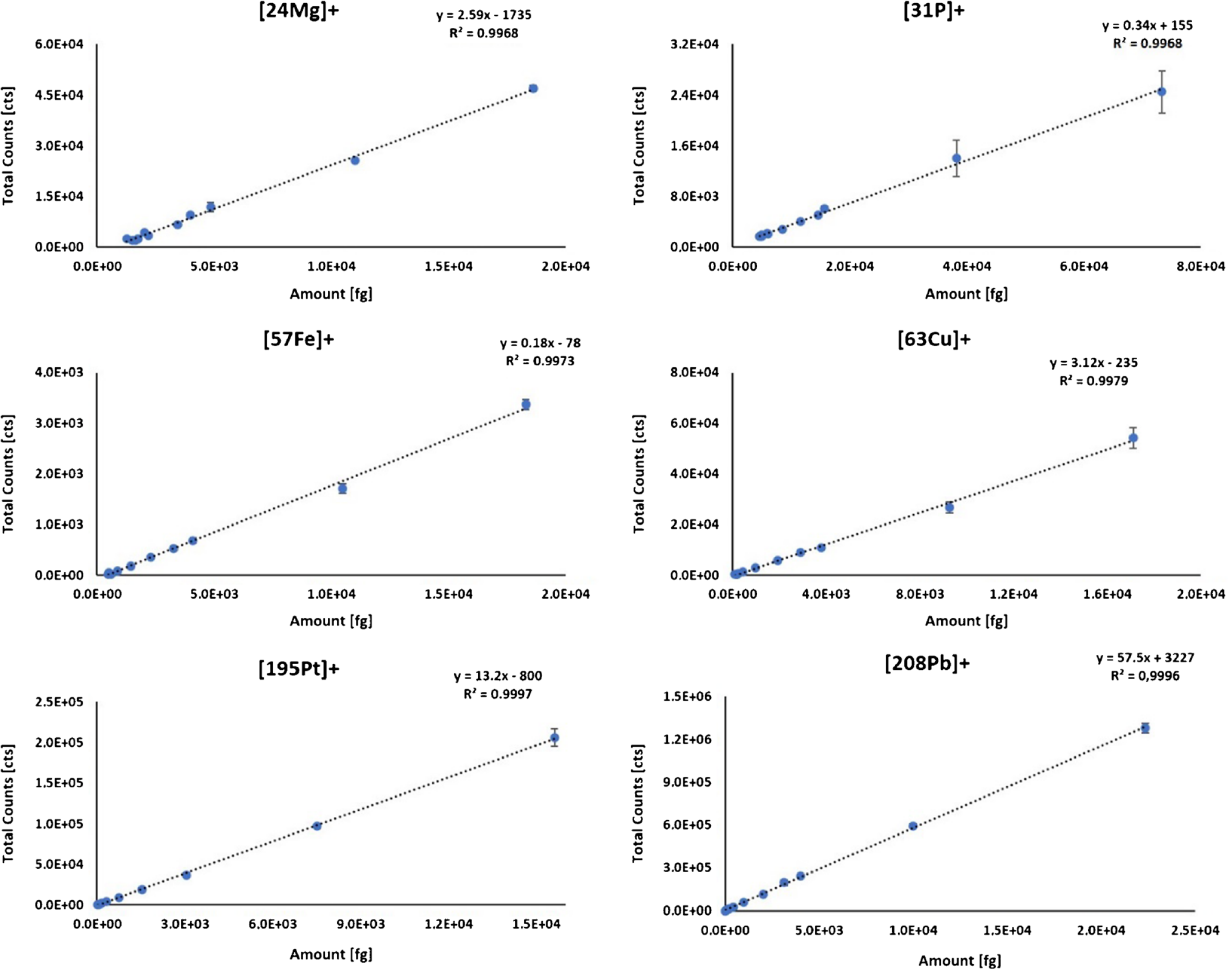
Calibration curves of selected elements using gelatin micro-droplet standards spiked with multi-element standard solutions. The standards were measured in standard mode by LA-ICP-TOFMS. Each standard concentration was measured four times

Elemental quantities in the prepared gelatin standards were experimentally assessed by solution-based ICP-MS following acid-assisted digestion of the standards. The summed signal intensity for each deposited micro-droplet standard was calculated using HDIP by creating volumes of interest (VOIs) of the droplets and the built-in feature to calculate the sum of the VOIs. The corresponding droplet volumes (determined by the micro-spotter) were used to calculate the analyte amounts for the integrated signal response of the standards. Calibration curves of different elements measured in standard mode by LA-ICP-TOFMS are shown in Fig. 1 and ESM Fig. S1. A good linear fit was obtained for the elements shown. The calibrations were linear over two orders of magnitude (depending on the element, between 50 and 200 fg up to 20 pg) which corresponded to concentrations ranging from 0.1/0.5 to 50 μg g^−1^. Each concentration level was measured in replicates (*n* = 4) with experimental uncertainties ranging at about 5–15%, depending on the element and concentration level.

Absolute limits of detection (LODs) on pixel base are reported in Table 1 for a wide range of elements using the gelatin micro-droplet standards and LA-ICP-TOFMS detection in standard mode and in CCT mode, where the collision/reaction cell was pressurized with a mixture of H_2_/He gas. Limits of detection were calculated according to Longerich et al. [32] for individual pixels with a pixel size of 2.5 μm × 2.5 μm. The use of the CCT mode significantly improved the LOD of ^56^Fe due to the higher mass resolving power and suppression of the ^40^Ar^16^O signal.

**Table 1:** Absolute limits of detection for selected elements using gelatin micro-droplet standards and LA-ICP-TOFMS detection in standard mode and in H_2_/He gas (CCT) mode. LODs are calculated per pixel based on Longerich et al. [32]

	Limits of detection			
	Standard mode		CCT mode	
	Absolute amount (fg per pixel)	Concentration (μg g^−1^ per pixel)	Absolute amount (fg per pixel)	Concentration (μg g^−1^ per pixel)
^24^Mg	17.9	573	0.28	8.95
^31^P	3.8	122	11.9	381
^49^Ti	0.18	5.87	0.071	2.27
^51^V	0.006	0.18	0.015	0.47
^52^Cr	0.28	8.89	0.036	1.14
^55^Mn	0.017	0.56	0.02	0.65
^56^Fe	9.93	318	0.41	13.0
^57^Fe	3.33	107	8.95	286
^59^Co	0.003	0.11	0.0006	0.02
^60^Ni	0.014	0.45	0.020	0.65
^63^Cu	0.28	9.06	0.032	1.03
^65^Cu	0.64	20.6	0.074	2.36
^64^Zn	0.38	12.3	0.020	0.64
^66^Zn	0.39	12.5	0.007	0.23
^75^As	0.036	1.15	0.026	0.82
^95^Mo	0.002	0.05	0.0001	0.004
^101^Ru	0.001	0.04	0.001	0.03
^114^Cd	10.4	333	0.007	0.22
^121^Sb	0.07	2.28	0.0006	0.019
^137^Ba	0.31	10.0	0.031	0.98
^195^Pt	0.0002	0.007	0.0002	0.0066
^205^Tl	0.001	0.029	0.00003	0.0009
^208^Pb	0.024	0.76	0.0012	0.039

### Accuracy of the gelatin micro-droplet quantification approach

As the availability of certified reference materials for bioimaging applications by LA-ICPMS is limited, the accuracy of the developed calibration approach was evaluated using human plasma samples spiked with varying concentrations of P, Fe, Cu, Zn, and Pt standard solutions and flow injection (FI)-ICP-MS/MS analysis. Subsequently, the spiked plasma samples were spotted onto glass slides similar to the gelatin micro-droplets resulting in droplets of around 200 μm in diameter. The entire plasma droplets were quantitatively ablated and elemental concentrations were determined using the gelatin micro-droplets and LA-ICP-TOFMS detection. In parallel, the spiked plasma samples were analyzed by FI-ICP-MS/MS in standard mode and in oxygen gas mode and the concentrations were determined using liquid multi-element standards. The results of the flow injection ICP-MS/MS measurements were used as reference values for the LA-ICP-TOFMS analysis (Table 2). The accuracy of the FI-ICP-MS/MS method was determined using the serum reference material Seronorm. For quantification by LA-ICP-TOFMS, Seronorm reference material could not be used to determine the accuracy of Fe, Cu, and Zn as the reference material had to be diluted by a factor of 4 for micro-droplet spotting due to its high protein content. Due to the dilution, the concentrations of Fe, Cu, and Zn in Seronorm were under the limit of detection of the LA-ICP-TOFMS method.

**Table 2:** Multi-element quantification in spiked human plasma samples using LA-ICP-TOFMS detection and gelatin micro-droplets as calibration standards, for *n* = 4 samples. Flow injection in combination with ICP-MS/MS detection was used as a complementary method for *n* = 4 samples and the determined concentrations were used as reference values. The confidence level was 95%

	FI-ICP-MS/MS		LA-ICP-TOFMS				
	Concentration (μg L^−1^)	Confidence interval	Concentration (μg L^−1^)	Confidence interval	RSD (%)	Recovery (%)	Sample
^31^P	25.8 ± 0.24	0.38	26.4 ± 0.94	1.5	3.6	102	Human plasma
	27.9 ± 0.44	0.70	27.9 ± 0.49	0.78	1.8	100	Spiked human plasma 1
	30.4 ± 0.24	0.39	29.5 ± 1.3	2.1	4.4	97	Spiked human plasma 2
	40.3 ± 0.58	0.92	40.9 ± 0.88	1.4	2.2	101	Spiked human plasma 3
^31^P	54.2 ± 10.9^*^	-	49.6 ± 2.3	3.6	4.6	91	Seronorm
^63^Cu	0.31 ± 0.004	0.007	< LOD	-	-	-	Human plasma
	0.94 ± 0.004	0.007	0.92 ± 0.072	0.11	7.8	97	Spiked human plasma 1
	1.5 ± 0.04	0.07	1.37 ± 0.08	0.12	5.7	91	Spiked human plasma 2
	6.1 ± 0.06	0.1	5.2 ± 0.26	0.41	4.9	84	Spiked human plasma 3
^65^Cu	0.30 ± 0.004	0.007	< LOD	-	-	-	Human plasma
	0.92 ± 0.005	0.008	0.96 ± 0.05	0.08	4.9	104	Spiked human plasma 1
	1.5 ± 0.01	0.011	1.44 ± 0.06	0.1	4.4	94	Spiked human plasma 2
	6.2 ± 0.01	0.016	5.24 ± 0.22	0.35	4.2	84	Spiked human plasma 3
^64^Zn	0.17 ± 0.001	0.001	< LOD	-	-	-	Human plasma
	0.67 ± 0.004	0.006	0.65 ± 0.03	0.05	5.1	96	Spiked human plasma 1
	1.17 ± 0.01	0.021	1.13 ± 0.03	0.004	2.2	97	Spiked human plasma 2
	4.87 ± 0.05	0.073	4.61 ± 0.24	0.38	5.2	95	Spiked human plasma 3
^66^Zn	0.17 ± 0.001	0.002	< LOD	-	-	-	Human plasma
	0.69 ± 0.004	0.006	0.66 ± 0.007	0.01	1.1	95	Spiked human plasma 1
	1.17 ± 0.02	0.023	1.09 ± 0.06	0.09	5.3	92	Spiked human plasma 2
	5.0 ± 0.03	0.045	4.52 ± 0.30	0.47	6.6	90	Spiked human plasma 3
^195^Pt	0.54 ± 0.005	0.01	0.48 ± 0.02	0.04	5.1	90	Spiked human plasma 1
	1.04 ± 0.03	0.04	0.96 ± 0.04	0.06	4	93	Spiked human plasma 2
	5.14 ± 0.08	0.13	4.71 ± 0.20	0.32	4.3	92	Spiked human plasma 3

*Phosphor concentrations in Seronorm serum reference material; the target value provided by the manufacturer is used as reference value

Phosphorus, iron, copper, and zinc were selected, as they represent elements with major biological key functions from the lower mass range that are prone to interferences. The LA-ICP-TOFMS method provided an accurate quantification of phosphorus in spiked plasma samples at concentrations of 25, 30, and 40 μg L^−1^ and in Seronorm reference material (Table 2). In addition, the accurate quantification of ^63^Cu, ^65^Cu, ^64^Zn, and ^66^Zn was shown in spiked human plasma samples at concentrations of 1, 1.5, and 5 μg L^−1^ by the developed LA-ICP-TOFMS calibration method (Table 2). The results are within the confidence interval of the values determined by FI-ICP-MS/MS. For iron, the concentrations of the spiked plasma samples were under the limit of detection and could therefore not be determined. Platinum concentrations determined by LA-ICP-TOFMS in spiked plasma samples were in accordance with the concentrations determined by flow injection ICP-MS/MS for Pt concentrations of 0.5 μg L^−1^, 1 μg L^−1^, and 5 μg L^−1^ (Table 2). The validation of the calibration approach for platinum and for elements from the lower mass range proves that the developed calibration approach is fit-for-purpose for bioimaging applications by LA-ICP-(TOF)MS.

### Application of the developed calibration approach to multicellular tumor spheroids

**Fig. 2:**
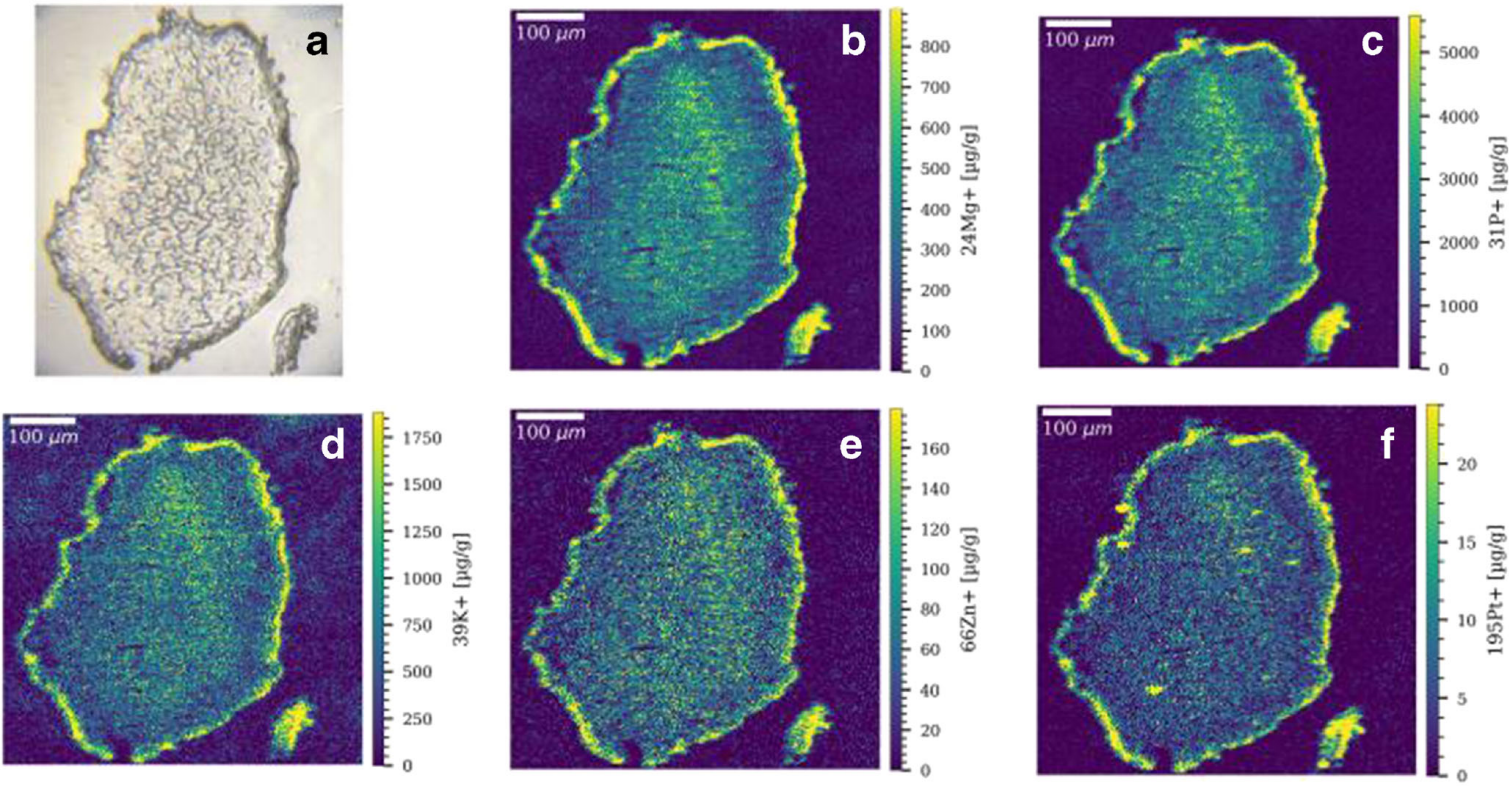
**a** Microscopic image of a colon cancer HCT116 tumor spheroid. Quantitative LA-ICP-TOFMS elemental images of **b** ^24^Mg^+^, **c** ^31^P^+^, **d** ^39^K^+^, **e** ^66^Zn^+^, and **f** ^195^Pt^+^ in a selected HCT116 tumor spheroid section after treatment with 20 μM cisplatin for 12 h. The following laser parameters were used: 5 μm × 5 μm square spot, fixed dosage mode 2, repetition rate: 200 Hz. The parallel line scans overlapped one another by 2.5 μm

**Fig. 3:**
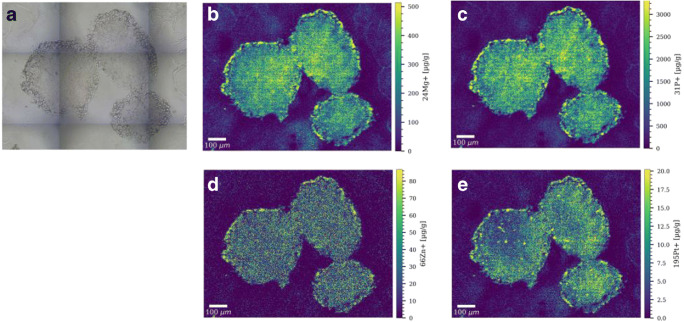
**a** Microscopic image of three colon cancer HCT116 tumor spheroids. Quantitative LA-ICP-TOFMS elemental images of **b** ^24^Mg^+^, **c** ^31^P^+^, **d** ^66^Zn^+^, and **e** ^195^Pt^+^ in selected HCT116 tumor spheroid sections after treatment with 20 μM oxaliplatin for 24 h. The following laser parameters were used: 5 μm × 5 μm square spot, fixed dosage mode 2, repetition rate: 200 Hz. The parallel line scans overlapped one another by 2.5 μm

As case study, the gelatin micro-droplet calibration approach was applied to the analysis of multicellular tumor spheroids treated with the Pt(II) anticancer drugs cisplatin and oxaliplatin at clinically relevant concentrations. Tumor spheroids are advanced in vitro models that recapitulate tumor tissue–like features and that are characterized by gradients in pH value, nutrients, oxygen, and metabolites leading to the formation of proliferating, quiescent, hypoxic, and necrotic regions [33–35]. The quantitative distribution maps are shown for selected elements in thin sections of multicellular HCT116 colorectal tumor spheroids in Figs. 2 and 3, and in ESM Figs. S3 and S4. Quantification was performed for several elements with biological key functions, including Mg, P, K, and Zn, and for Pt resulting from the Pt(II) anticancer drug treatment. The levels of Cu in the tumor spheroids were below the limits of detection of the method; therefore, only qualitative distribution maps of Cu are shown in ESM Fig. S5. For HCT116 tumor spheroids treated with 20 μM cisplatin for 12 h, a pronounced accumulation for Mg, P, K, Cu, Zn, and Pt was observed in the outer rim of the spheroids (Fig. 2 and ESM Fig. S4A). It is known that the outer rim of tumor spheroids is composed of proliferating cells, resembling cells in the tumor microenvironment that are close to blood vessels. The middle part of the tumor spheroid is composed of quiescent cells, which show a lower and relatively homogeneous distribution of platinum and the detected elements from the lower mass range. The elemental distribution pattern of Pt and P is in accordance with previous LA-ICPMS studies on tumor spheroids treated with oxaliplatin and Pt(IV) compounds [36, 37]. Elemental concentrations are about 2 to 3 times lower in the core of the spheroid compared to the proliferating cells. For platinum, a mean concentration of around 6.6 μg g^−1^ was calculated for the volume of interest of the core of the spheroid, compared to around 12 μg g^−1^ for the VOI containing proliferating cells. Tumor spheroids treated with 20 μM cisplatin for 24 h showed a relatively homogeneous distribution for Mg, P, Zn, and Pt with a small rim of proliferating cells with enhanced elemental levels (ESM Fig. S3). For platinum, some hotspots are visible in the middle of the spheroid section. As observed in the immunofluorescence image (ESM Fig. S6B), even in deeper cell layers of the spheroids, proliferating cells can be found, which might explain the presence of platinum hotspots. The platinum concentration showed a mean value of around 7.1 μg g^−1^ for this spheroid section. For the oxaliplatin-treated spheroid samples, a similar elemental pattern was observed in the spheroid sections with higher concentrations in the proliferating cells compared to the quiescent cells (Fig. 3 and ESM Fig. S4). Oxaliplatin treatment resulted in higher platinum concentrations in the HCT116 tumor spheroids compared to cisplatin (both drugs are given at equimolar concentration). Oxaliplatin is more active in colorectal tumors than cisplatin and there might be underlying mechanisms in their mode of action that possibly induce a higher uptake of oxaliplatin by this cell line in comparison to cisplatin [38]. In long-term incubation experiments, oxaliplatin induced more pronounced changes in the morphology (apoptotic shape) and in the size of the spheroids compared to cisplatin treatment (ESM Fig. S6A).

The developed gelatin micro-droplet calibration approach can be used to perform concentration- and time-dependent uptake studies of metal-based anticancer drug candidates in multicellular tumor spheroids and can be further applied to in vivo samples. The analysis of calibration standards, quality controls, and five tumor spheroids with a pixel size of 2.5 μm would require an LA-ICPMS analysis time of around 2 h with a pixel acquisition rate of 200 Hz.

## Conclusions

A quantification strategy based on gelatin micro-droplets was presented for multi-element analysis in biological samples using LA-ICP-TOFMS. The primary advantages of the developed method are the fully automatized and flexible production of calibration standards using gelatin as matrix and a micro-spotter, providing the necessary reproducibility of the micro-droplets. The small dimensions of the micro-droplet standards (around 200 μm in diameter) and the use of a low-dispersion LA system resulted in an analysis time of less than 5 min per standard, which is comparable to solution-based ICP-MS analysis. The accuracy of the calibration method was assessed by the analysis of spiked human plasma droplets by flow injection ICP-MS/MS as a complementary method. Using the proposed type of calibration standards, μg g^-1^-level concentrations of Mg, P, Zn, and Pt were evaluated at a pixel size of 2.5 μm in multicellular tumor spheroids after treatment with the anticancer drugs cisplatin and oxaliplatin. The proposed standards could be especially attractive in quantitative high-throughput single-cell analysis. Due to their facile production, the gelatin micro-droplet standards have the potential to improve intra- and inter-laboratory comparability for standard harmonization.

## Supplementary information

ESM 1 (DOCX 2370 kb)
