# Supplementary material for: Micro-droplet-based calibration for quantitative elemental bioimaging by LA-ICPMS
Source: Anal Bioanal Chem. 2021 May 5;414(1):485–95. doi: 10.1007/s00216-021-03357-w (PMC8748332; doi:10.1007/s00216-021-03357-w)
Supplement: Supplementary file 1 — (DOCX 2370 kb) [file 216_2021_3357_MOESM1_ESM.docx]

**Supplementary Information to**

**Micro-droplet-based calibration for quantitative elemental bioimaging by LA-ICPMS**

**Table S1: Instrumental parameters for ICP-MS measurements**

|  | ICP-MS | ICP-MS/MS | (LA-)ICP-TOFMS |
| --- | --- | --- | --- |
| Manufacturer | Agilent 7800 | Agilent 8800 | TOFWERK AG ICP-TOF2R |
| Plasma Power [W] | 1550 | 1550 | 1440 |
| Sampling depth [mm] | 6.3 | 7.8 | 3.5 |
| Cone materials | Ni | Ni | Ni |
| Plasma gas flow [L min^-1^] | 15 | 15 | 14 |
| Auxiliary gas flow [L min^-1^] | 0.90 | 0.80 | 0.80 |
| Nebulizer gas flow [L min^-1^] | 1.08 | 1.07 | 0.95 |
| Measurement modes | standard mode, He gas mode | standard mode,  O_2_ gas mode | standard mode,  H_2_/He gas mode |


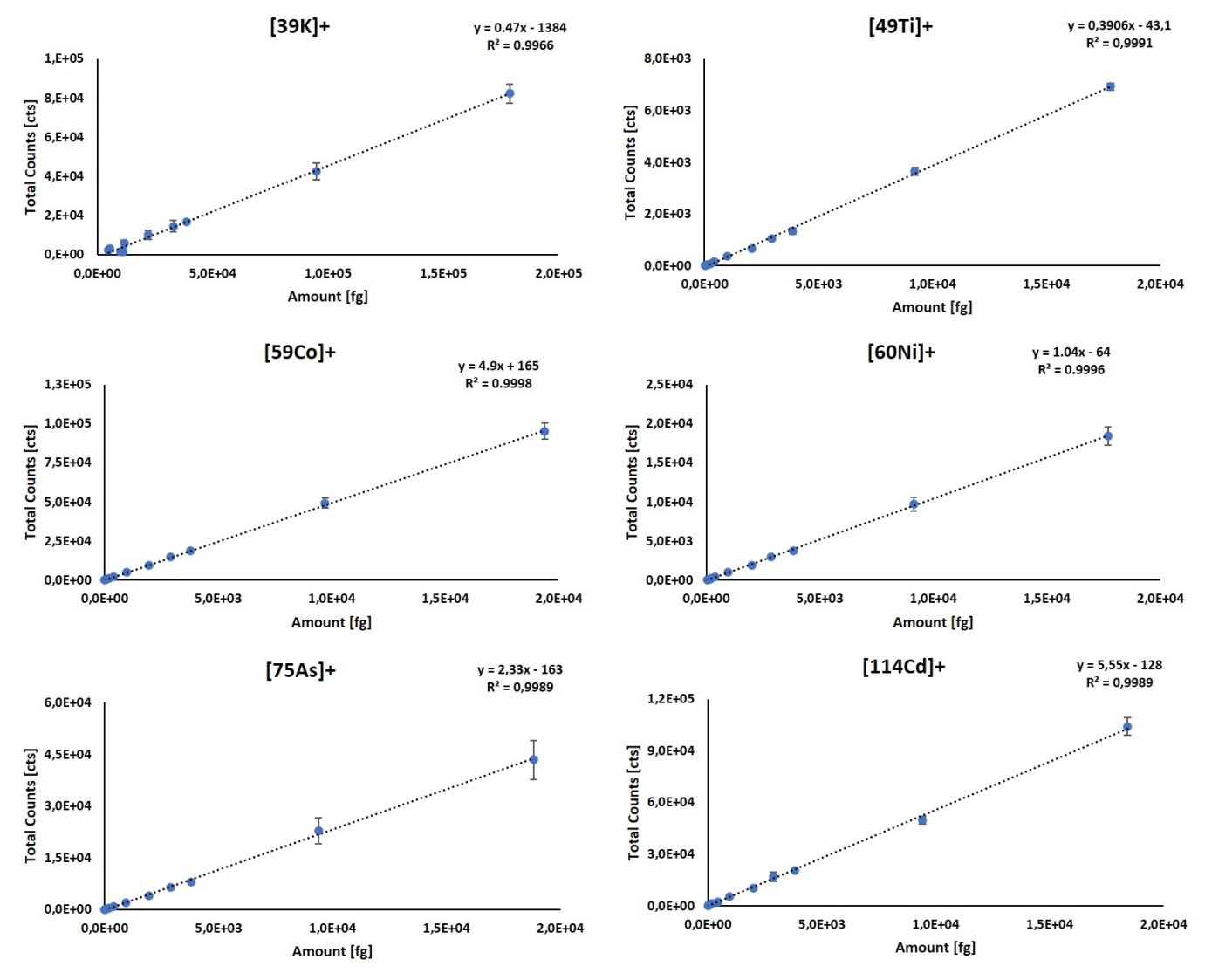


**Figure S1.** Calibration curves of selected elements using gelatin micro-droplet standards spiked with multi-element standard solutions and measured in standard mode by LA-ICP-TOFMS. Each standard concentration was measured four times.

**Equations used to calculate concentrations**

$m_{g std}=m_{g}+m_{std}+m_{sol}$ (Equation S1)

$w_{{g std}_{i}}=\frac{w_{{std}_{i}}\cdot m_{std}}{m_{g std}}$ (Equation S2)

$m_{g std}$ Mass of the gelatin standard mixture (g)

$m_{g}$ Mass of the gelatin (g)

$m_{std}$ Mass of the standard solution (g)

$m_{sol}$ Mass of the solvent (g)

$w_{{g std}_{i}}$ Mass fraction of element i within the gelatin standard mixture

$w_{{std}_{i}}$ Mass fraction of element i within the standard solution

$m_{j_{g std}}=V_{j_{g std}}\cdot\rho_{g std}$ (Equation S3)

$m_{i,j}=w_{{g std}_{i}}\cdot m_{j_{g std}}$ (Equation S4)

$m_{j_{g std}}$ Mass of the gelatin standard mixture droplet of concentration level j as created

by the micro-spotter (g)

$V_{j_{g std}}$ Volume of the gelatin standard mixture droplet of concentration level j as created

by the micro-spotter (ml)

$\rho_{g std}$ Density of the gelatin standard mixture (g ml^-1^)

$m_{i,j}$ Mass of element i in droplet of concentration level j (g)

$w_{{g std}_{i}}$ Mass fraction of element i within the gelatin standard mixture

$A_{i,j}=\frac{R_{i,j}}{m_{i,j}}$ (Equation S5) $A_{i}=n^{-1}\sum_{j=1}^{n} A_{i,j}$ (Equation S6)

$A_{i,j}$ Absolute sensitivity of element i at concentration level j (cts g^-1^)

$R_{i,j}$ Integrated response of element i at concentration level j (cts)

$A_{i}$ Average sensitivity of element i (cts g^-1^)

$n$ Number of concentration levels

$m_{p}=\delta_{t}\cdot a^{2}\cdot\rho_{t}$ (Equation S7)

$w_{i,p}=\frac{\frac{R_{i,p}}{A_{i}}}{m_{p}}$ (Equation S8)

$m_{p}$ Ablated mass per pixel p related to the fresh sample (g)

$\delta_{t}$ Thickness of the fresh sample t (cm)

$a^{2}$ Ablated area per pixel (cm²)

$\rho_{t}$ Density of the fresh sample t (g ml^-1^)

$w_{i,p}$ Mass fraction of element i in pixel p

$R_{i,p}$ Integrated response of element i (cts)


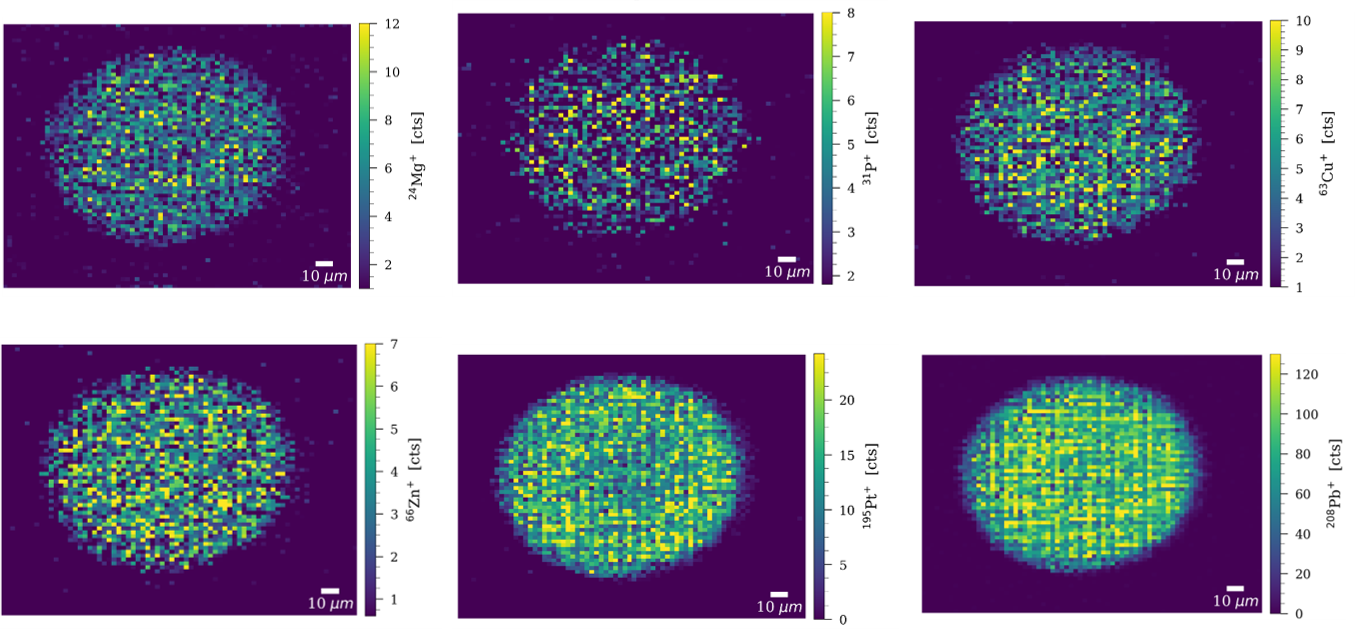


**Figure S2.** Elemental distribution maps of selected elements in gelatin micro-droplet standards. The following laser parameters were used: spot size: 5 µm square, fixed dosage mode 2, repetition rate: 200 Hz. The parallel line scans overlapped one another by 2.5 µm.


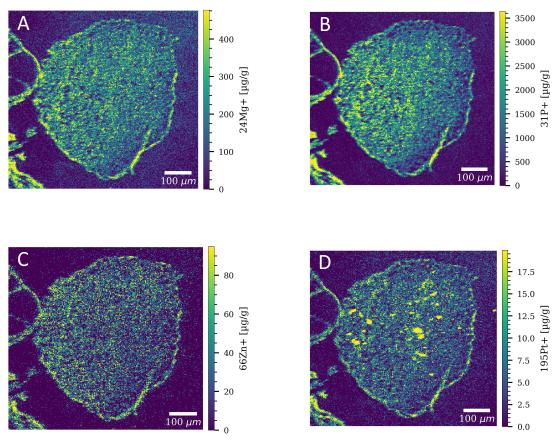


**Figure S3.** Quantitative LA-ICP-TOFMS elemental images of (A) ^24^Mg^+^, (B) ^31^P^+^, (C) ^66^Zn^+^ and (D) ^195^Pt^+^ in a selected HCT116 tumor spheroid section after treatment with 20 µM cisplatin for 24 h. The following laser parameters were used: spot size: 5 µm square, fixed dosage mode 2, repetition rate: 200 Hz. The parallel line scans overlapped one another by 2.5 µm.


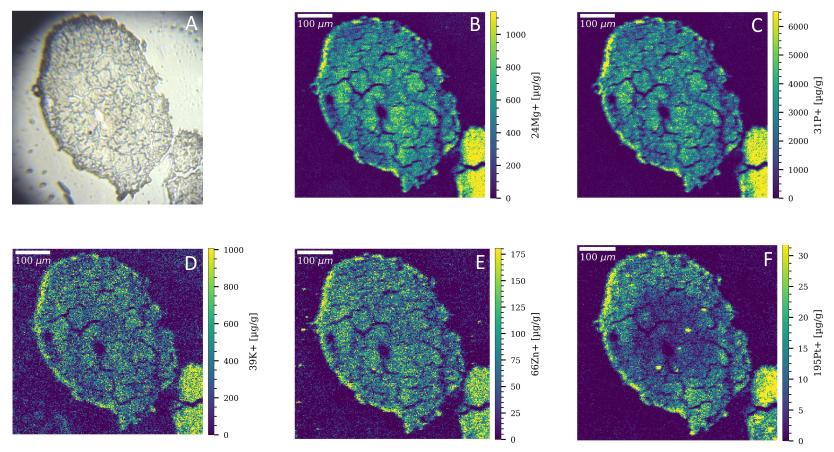


**Figure S4.** (A) Microscopic image of a colon cancer HCT116 tumor spheroid. Quantitative LA-ICP-TOFMS elemental images of (B) ^24^Mg^+^, (C) ^31^P^+^, (D) ^39^K^+^, (E) ^66^Zn^+^ and (F) ^195^Pt^+^ in a selected HCT116 tumor spheroid section after treatment with 20 µM oxaliplatin for 12 h. The following laser parameters were used: spot size: 5 µm square, fixed dosage mode 2, repetition rate: 200 Hz. The parallel line scans overlapped one another by 2.5 µm.


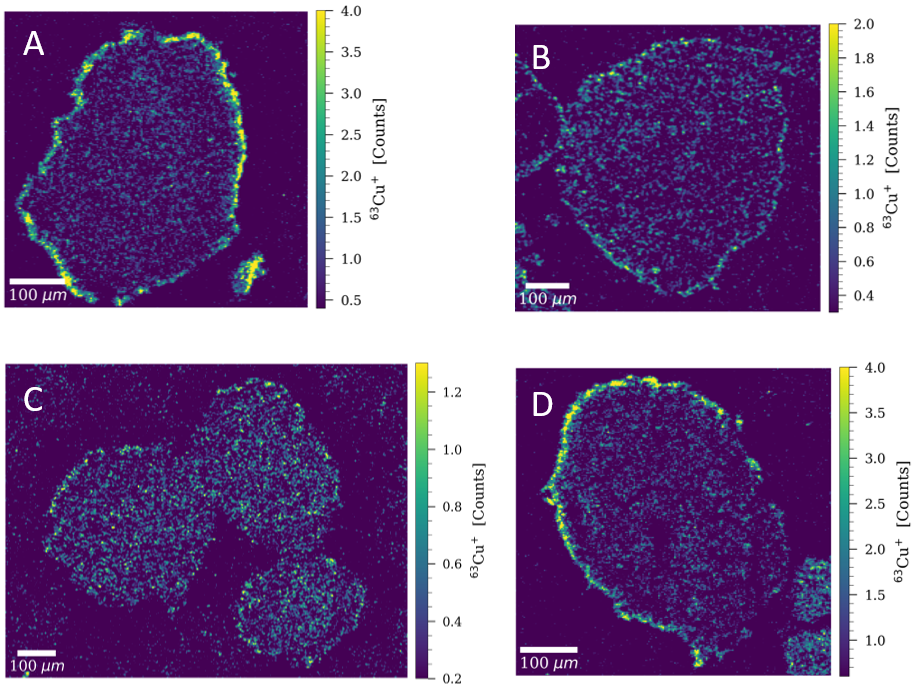


**Figure S5.** LA-ICP-TOFMS images of the ^63^Cu^+^ distribution in selected HCT116 tumor spheroid sections after treatment with (A) 20 µM cisplatin for 12 h, (B) 20 µM cisplatin for 24 h, (C) 20 µM oxaliplatin for 24 h and (D) 20 µM oxaliplatin for 12 h . The following laser parameters were used: spot size: 5 µm square, fixed dosage mode 2, repetition rate: 200 Hz. The parallel line scans overlapped one another by 2.5 µm.

**
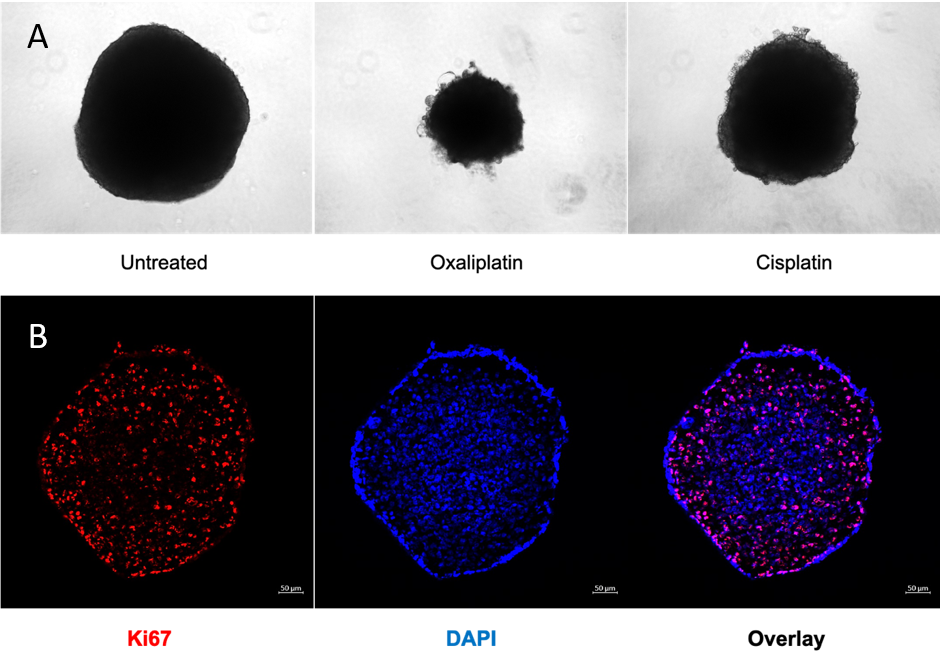
**

**Figure S6**: (A) Representative pictures of HCT116 multicellular spheroids treated with oxaliplatin and cisplatin for 11 days. Oxaliplatin induced more pronounced changes in the morphology (apoptotic shape) and in the size of the spheroids compared to cisplatin treatment. (B) Representative immunofluorescence analysis (confocal microscopy) of paraformaldehyde-fixed (PFA) HCT116 spheroids immuno-labelled with the cell proliferation marker Ki67. KI67^+^ cells are distributed within the whole spheroid, however they are less abundant in the core of the spheroids. Scale bar = 50 µm.
